# On the Preparation of Anatomical Material

**Published:** 1896-08

**Authors:** 


### ON THE PREPARATION OF ANATOMICAL MATERIAL.

At the recent meeting of the Association of American Anatomists a committee composed of Drs. Mears, Bryant, and Dwight made an interesting report upon the subject of the preservation of anatomical material. The keeping of bodies by means of cold storage, a plant costing from five hundred to three thousand dollars, is believed to be the method which approaches most nearly to perfection. The bodies thus preserved should be injected before being placed in the cold storage. It is suggested that in large cities, where several institutions exist, one plant would suffice. Of all the anatomical laws upon the statute books of the various States, that of Pennsylvania is considered to give the most satisfactory results. One novel method of keeping the subject is as follows: Inject with carbolic acid, one and one-half pints; glycerin, six pints; and alcohol, one and one-half pints. After the injection has been made the subject should be painted daily for fourteen days with one part carbolic acid to six parts of glycerin. It is then to be placed in an air-tight box over a jar of methylated spirits. By this method there is said to be an absence of odor from the body and a characteristic appearance of the tissues. Wickersheim's formula consists of three thousand parts of pure water, one hundred and nine parts of alum, twenty-five parts of chloride of sodium, twelve parts of nitrate of potassium, sixty parts of carbonate of potassium, and ten parts of arsenious acid; to ten parts of a liquid so prepared one part of methylic alcohol and four parts of glycerin are added. Formalin may be used in the proportion of from one-half to one per cent., though the odor is disagreeable, and the effect upon the skin of the hand is annoying.—*International Medical Annual.*

